# The circadian clock is associated with prognosis and immune infiltration in stomach adenocarcinoma

**DOI:** 10.18632/aging.203184

**Published:** 2021-06-23

**Authors:** Zhihao Huang, Aoxiao He, Jiakun Wang, Hongcheng Lu, Rongguiyi Zhang, Linquan Wu, Qian Feng

**Affiliations:** 1 Department of General Surgery, The Second Affiliated Hospital of Nanchang University, Nanchang 330000, China; 2 Department of Emergency, The Second Affiliated Hospital of Nanchang University, Nanchang 330000, China

**Keywords:** stomach adenocarcinoma, bioinformatics analysis, cancer immunotherapy, circadian clock

## Abstract

Background: Stomach adenocarcinoma (STAD) is one of the most prevalent malignances and ranks fifth in incidence and third in cancer-related death among all malignances. The prognosis of STAD is poor. The circadian clock is regulated by interlocked transcriptional-translational feedback loops that orchestrate circadian rhythms in some biological processes, including the immune response and metabolism. However, the association between core circadian clock genes and STAD patient prognosis is unclear.

Materials and Methods: In our study, bioinformatics methods were performed to explore the expression and prognostic value of core circadian clock genes in STAD and their association with immune infiltration.

Results: The mRNA levels of CLOCK, CRY1 and NR1D1 were upregulated, while the mRNA levels of CRY2, PER1, PER3 and RORA were downregulated in STAD tissues compared with normal tissues. Core circadian clock genes exert promoting or inhibiting effects on certain cancer-related hallmark pathways, including the DNA damage response, cell cycle, apoptosis and RAS/MAPK pathways. Moreover, core circadian clock genes were linked to drug sensitivity or drug resistance. Prognosis analysis revealed that high expression of PER1 and NR1D1 was associated with poor overall survival, progression-free survival, and disease-free survival rates in STAD patients. Validation analysis further confirmed our result. Immune infiltration analysis demonstrated that the expression of ICOSLG and CD70 was significantly correlated with immune cells, immune biomarkers, chemokines and their receptors.

Conclusions: Our results suggest that NR1D1 and PER1 are prognostic biomarkers and are associated with immune infiltration in STAD.

## INTRODUCTION

Stomach adenocarcinoma (STAD) is a global health problem and ranks fifth in incidence and third in cancer-related death among all malignances [1, 2]. It is thought that there are approximately 1 million new STAD cases diagnosed each year globally [3]. Although some risk factors have been identified, including *Helicobacter pylori* infection, and targeted therapy drugs, including trastuzumab, have been used for the treatment of STAD, the prognosis of STAD patients is still poor, with an overall survival rate of approximately 12 months in patients with advanced-stage STAD [4]. Thus, it is urgent to explore new marker for the prognosis and therapy for STAD patients.

The circadian clock and circadian rhythms play a vital part in some biological processes, including the immune response and metabolism [5]. Immunotherapy is increasingly suggested as the most promising therapy for STAD, in addition to operative treatment, especially for patients with advanced STAD [6]. A previous study found a close association between the immune system and the circadian clock [7]. Cancer chronotherapy that treats cancer according to a specific time in the circadian rhythm can improve the effectiveness and safety of drugs by optimizing the duration of medication efficacy without increasing the dose or changing the drug type [5]. To date, several core circadian clock genes, including but not limited to CLOCK, BMAL1, CRY1, CRY2, NR1D1, PER1, PER2, PER3, and RORA, have been reported in the literature [5]. However, the potential function of circadian clock in patients’ prognosis and their clinical significance in STAD are still unclear.

Increasing evidence has indicated that genomic research is one of the most reliable methods to accelerate the clinical and translational research and treatment of cancer. Accumulating evidences have elucidated the significance of circadian clock in different types of cancer, including thoracic cancer, bladder cancer and glioma [8, 9]. However, how the circadian clock interacts with the tumor microenvironment and immune infiltrates in STAD is still unclear. Therefore, our research hope to elucidate the expression of circadian clock in STAD and their relationship with prognosis and immune infiltration, and to use a high-throughput sequencing database to propose more suitable strategies for improving the anti-immune performance of STAD.

## MATERIALS AND METHODS

### Data sets

We downloaded gene-expression data of STAD from The Cancer Genome Atlas (TCGA) (https://portal.gdc.cancer.gov/) on January 12, 2021, and the TCGA STAD dataset (*n* = 415) was isolated for analysis. Moreover, we also downloaded the clinical information of each STAD patient on January 12, 2021, such as sex, tumor grade and survival status. Gene expression between STAD and gastric tissue was analyzed with Student's *t*-test. A sanguini diagram was built based on the R software package ggalluvial. R software v4.0.3 was utilized to perform Statistical analyses with a *p*-value of 0.05 as the threshold value.

### Genetic mutation and pathway activity analysis

The “maftools” package was utilized to analyze and visualize genetic mutation data obtained from the TCGA database. Number of mutated sample/all STAD Sample were defined as the gene SNV percentage. The horizontal histogram showed the genes with higher mutation frequency in STAD patients. Pathway activity module of GSCALite, a TCGA visualization web portal, was used to detect the role in the pathway activity analysis. And the method was described before [10].

### Drug sensitivity analysis

To analyze the correlation between the gene expression of core circadian clock genes and drug sensitivity, we collected small molecules or drugs from the Therapeutics Response Portal (CTRP). We downloaded the area under the dose-response curve (AUC) values for drugs and gene expression profiles for all cancer cell lines. The correlation between gene expression with drug sensitivity (IC50) was explored with Spearman correlation analysis.

### Functional enrichment analysis

The differential expression of mRNAs with |Log (Fold Change)| >3 thresholds was firstly identified by using “limma” package. After that, the "ggplot2" package in R software was used to perform Gene Ontology (GO) analysis, including the biological process (BP), cellular component (CC), and molecular function (MF) categories. Moreover, this package was also utilized to perform Kyoto Encyclopedia of Genes and Genomes (KEGG) analysis.

### Survival analysis

The median gene expression was set as cut-off value to divide STAD cohort into two groups (high vs low). The survival difference was detected with a Kaplan-Meier analysis using TCGA STAD and GSE62254 dataset. Log-rank test was conducted to calculate the *p*-values and hazard ratios (HRs) with 95% confidence intervals (CIs). All analytical methods above were conducted with R software. Moreover, univariate and multivariate Cox regression analyses were conducted to find out the appropriate factors to construct a nomogram, which could be utilized to compute the prognostic risk for STAD patients.

### Validation of clinical tissues

After approved by the Ethics Committees of Second Affiliated Hospital of Nanchang University, STAD tissues (*n* = 46) and the corresponding gastric tissues (located 2 cm away from STAD lesions) were obtained from patients and each patient had received and told the informed consent. None of patients receive treatment before operation. Total RNA of tissues and qRT-PCR were conducted as described of our study before [11]. Supplementary Table 1 presented the primer sequences. The difference of PER1/NR1D1 expression and the prognosis of PER1/NR1D1 in STAD were evaluated with Student's *t*-test and Kaplan-Meier analysis.

### Immune infiltration

The correlation between the gene expression of core circadian clock genes and the abundances of six immune infiltrates (B cells, CD4+ T cells, CD8+ T cells, neutrophils, macrophages, and dendritic cells) was analyzed with TIMER (https://cistrome.shinyapps.io/timer/), a web portal for immune infiltration. Moreover, a two-sided Wilcoxon rank-sum test was performed to detect the difference of tumor infiltration levels affecting by different somatic copy number alterations.

## RESULTS

### Defining core circadian clock genes in STAD

**Figure 1:**
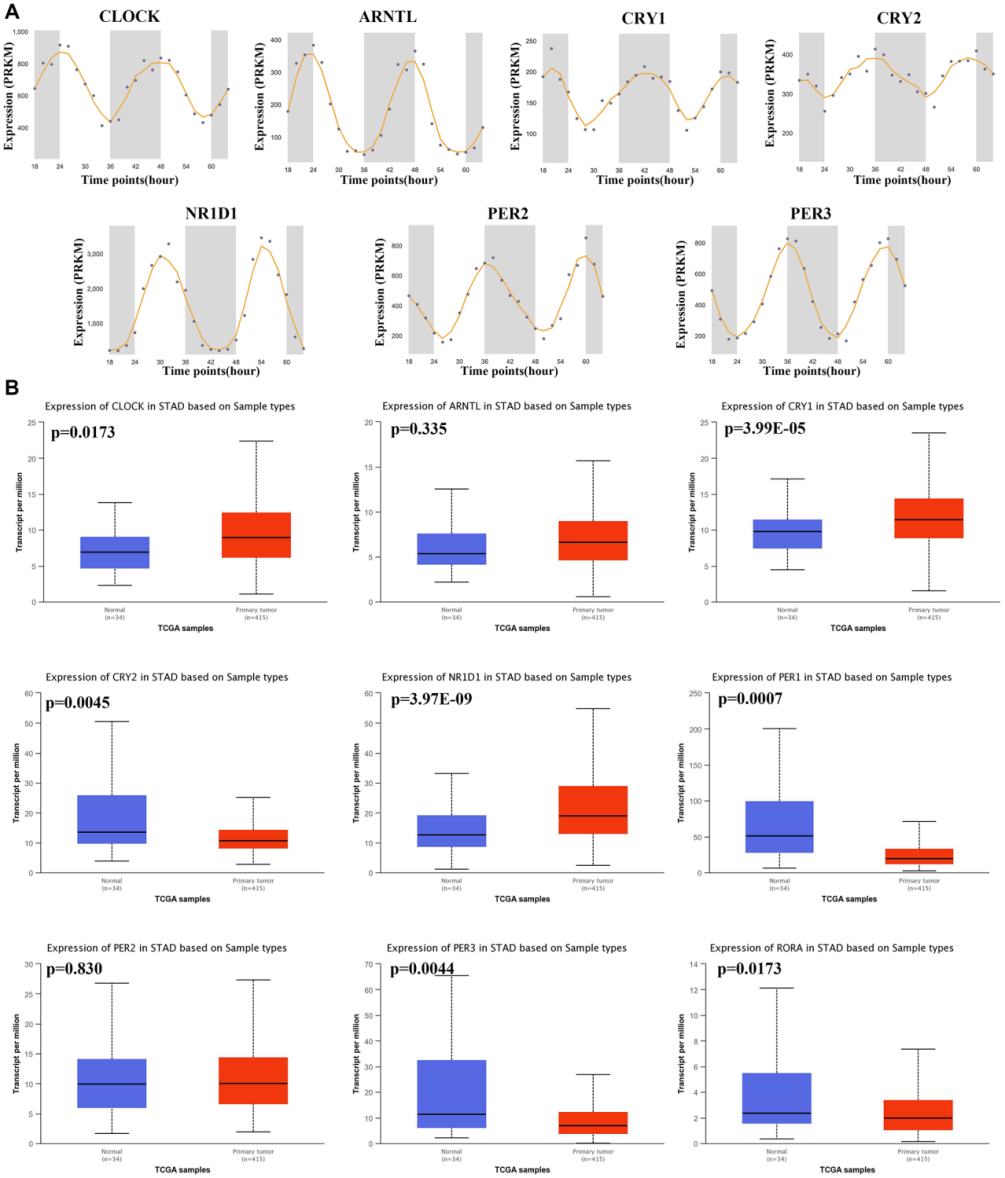
**The circadian rhythm and mRNA level of core circadian clock genes.** (**A**) The circadian rhythm of core circadian genes, including CLOCK(A), ARNTL(B), CRY1(C), CRY2(D), NR1D1(E), PER2(F) and PER3(G). (**B**) Box plots derived from TCGA STAD dataset comparing the expression of CLOCK, ARNTL, CRY1, CRY2, NR1D1, PER1, PER2, PER3 and RORA in STAD tissue and normal tissues.

Gene expression profile was used to explore the mRNA level of core circadian genes in gastric tissues at different times, and it was found that these genes showed oscillation at different times (Figure 1A). Using the TCGA STAD dataset, we then computed the mRNA level of these genes in STAD tissues and gastric tissues. The result is shown in Figure 1B. The mRNA levels of CLOCK (*p* = 0.0173), CRY1 (*p* = 3.99E-5) and NR1D1 (*p* = 3.97E-9) were upregulated, while the mRNA levels of PER1 (*p* = 0.0007), RORA (*p* = 0.0173), CRY2 (*p* = 0.0045) and PER3 (*p* = 0.0044) and were decreased in STAD tissues versus gastric tissues (Figure 1B). This evidence demonstrates that core circadian clock genes are widely altered at the mRNA level in STAD.

### Gene mutation landscape, hallmark pathways and drug sensitivity analysis

**Figure 2:**
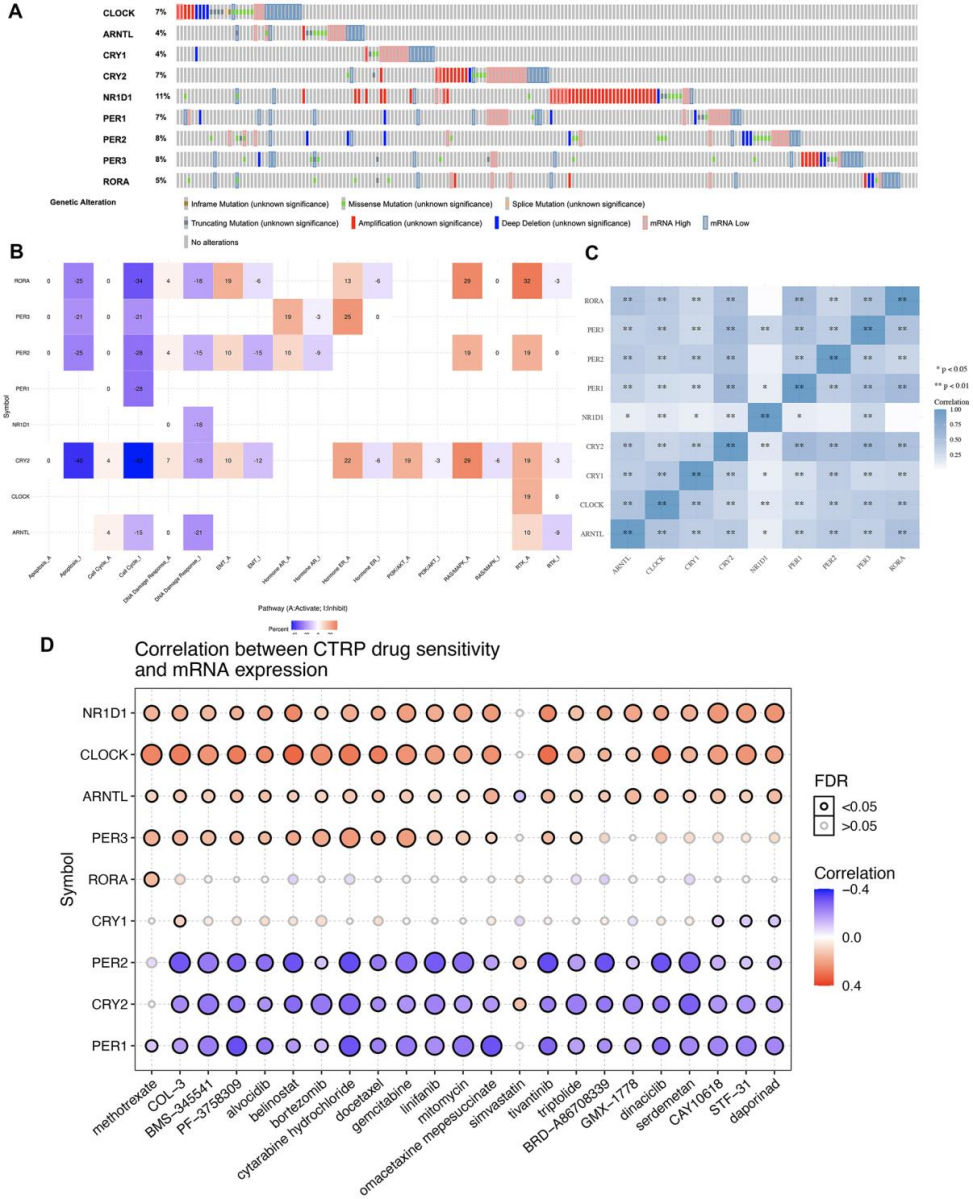
**Genetic mutation landscape and drug sensitivity analysis of core circadian clock genes in STAD.** (**A**) Oncoplot displaying genetic mutation landscape of core circadian clock genes in TCGA STAD cohort. (**B**) the role of circadian clock genes in the famous cancer related pathways. (**C**) A heat map of the correlation between each member of core circadian clock genes. (**D**) the correlation between core circadian clock genes and drug or small molecules. The Spearman correlation represent the core circadian clock genes expression correlates with the drug. The positive correlation means that the gene high expression is resistant to the drug, vise verse. ^*^p < 0.05, ^**^p < 0.01.

The gene mutation landscape of core circadian clock genes is presented in Figure 2. The results showed that genetic mutations of core circadian clock genes included inframe_mutation, Missense_nutation, splice_mutation, truncating mutation, amplification, deep deletion, mRNA high and mRNA low (Figure 2A). Among these genes, NR1D1 was the most mutated core circadian clock gene, with a genetic mutation rate of 11% in the TCGA STAD cohort (Figure 2A). To elaborate underlying functions of disorder of the circadian clock, our study then analyzed association between core circadian clock genes and well-known cancer-related hallmark pathways in STAD. We found that core circadian genes exerted inhibitory effects on the apoptosis pathway, cell cycle pathway, and DNA damage response pathway (Figure 2A). Moreover, certain circadian genes exerted promoting effects on the RAS/MAPK pathway and the RTK pathway (Figure 2B). Correlation analysis suggested a positive correlation between each member of the core circadian genes (Figure 2C). To verify whether the core circadian genes could serve as therapeutic targets, it is important to evaluate their association with existing drug targets. Clock-related drug administration at specific times during circadian cycles may remarkably improve the effectiveness of the drug and reduce the toxicity. In our study, drug sensitivity analysis revealed that the expression of PER2, CRY2 and PER1 was linked to drug sensitivity (negative correlation), while the expression of NR1D1, CLOCK, PER3 and ARNTL was linked to drug resistance (positive correlation) (Figure 2D).

### Enrichment analysis of differentially expressed genes (DEGs) in STAD

**Figure 3:**
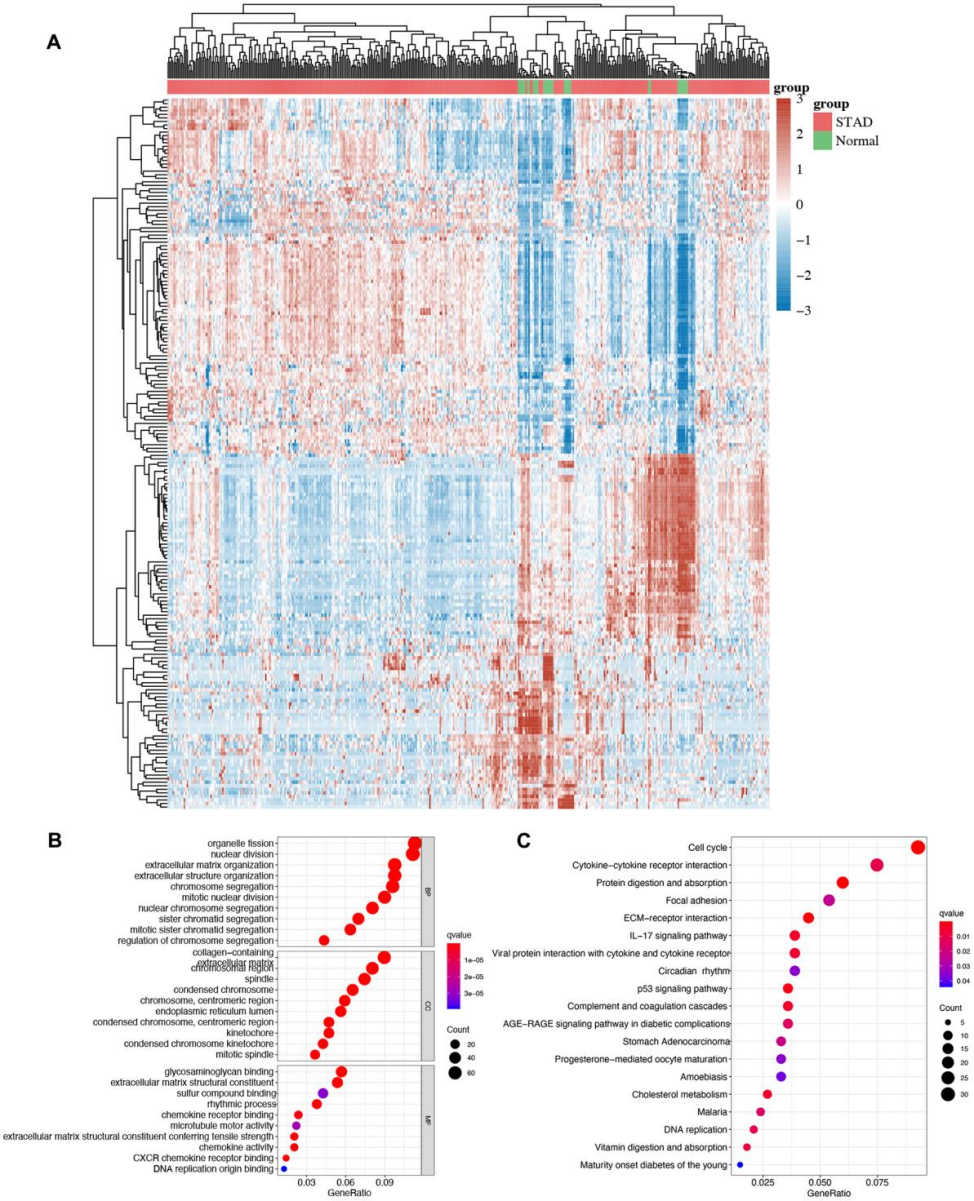
**The enrichment analysis of different expression genes in STAD.** (**A**) Heatmap of different expression genes in STAD. (**B**)The enriched items in GO analysis. (**C**) The enriched items in KEGG analysis.

We then performed GO and KEGG analyses using DEGs and the heatmap of DEGs in STAD is shown in Figure 3A. GO analysis demonstrated the correlation of these DEGs in organelle fission, nuclear division, mitotic nuclear division, condensed chromosome, glycosaminoglycan binding, rhythmic process, and chemokine activity (Figure 3B). Moreover, KEGG pathway demonstrated that these DEGs were primarily enriched in the cell cycle, cytokine-cytokine receptor interaction, circadian rhythm, p53 signaling pathway, STAD and DNA replication (Figure 3C).

### Prognosis value of core circadian genes in STAD

**Figure 4:**
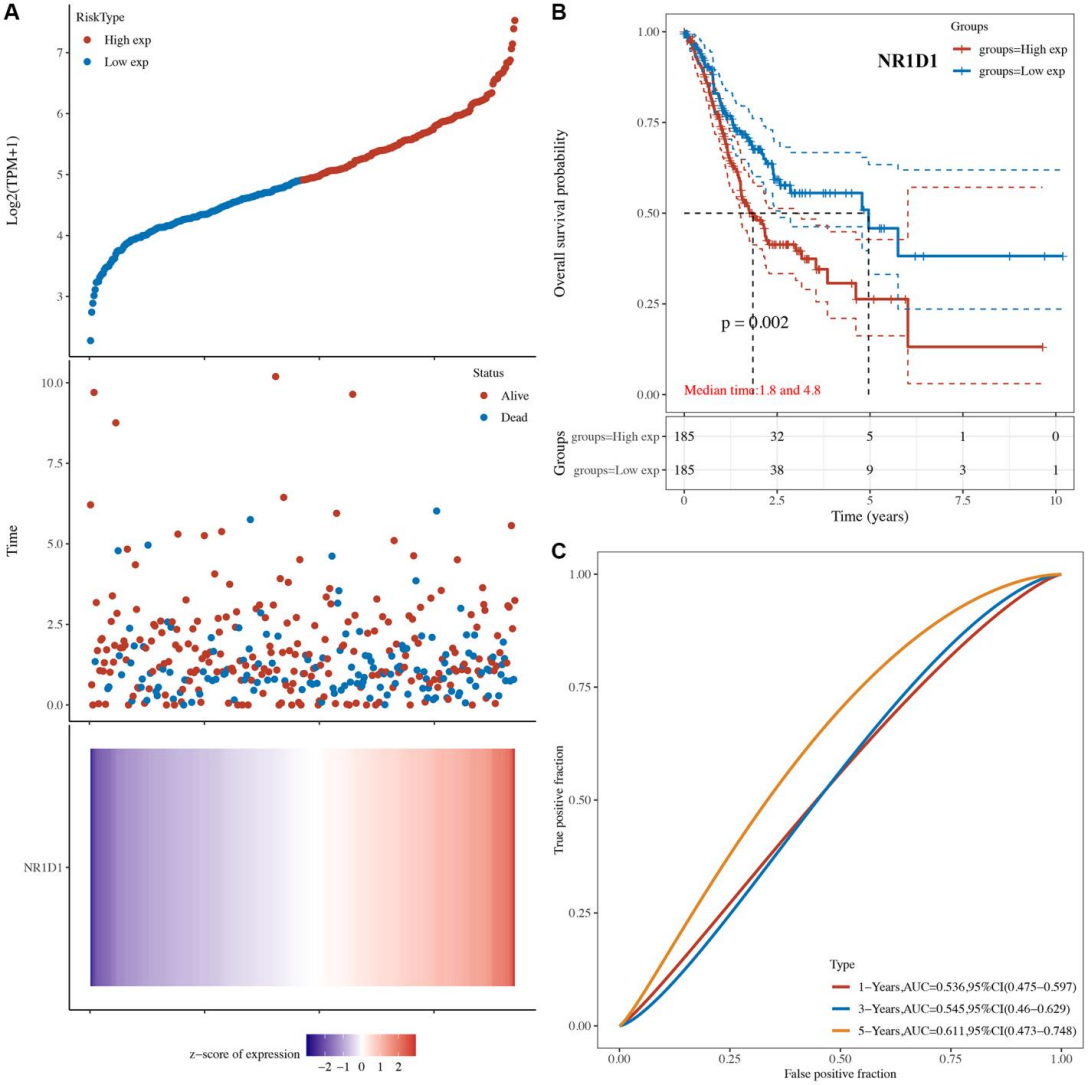
**The overall survival analysis of NR1D1 in STAD.** (**A**) The risk score, survival status and gene expression of each patient. (**B**) Kaplan-Meier overall survival curve of NR1D1 in STAD patients with high and low NR1D1 expression. (**C**) Time-dependent ROC of NR1D1 in predicting the prognosis of STAD patients. ROC receiver operating characteristic.

**Figure 5:**
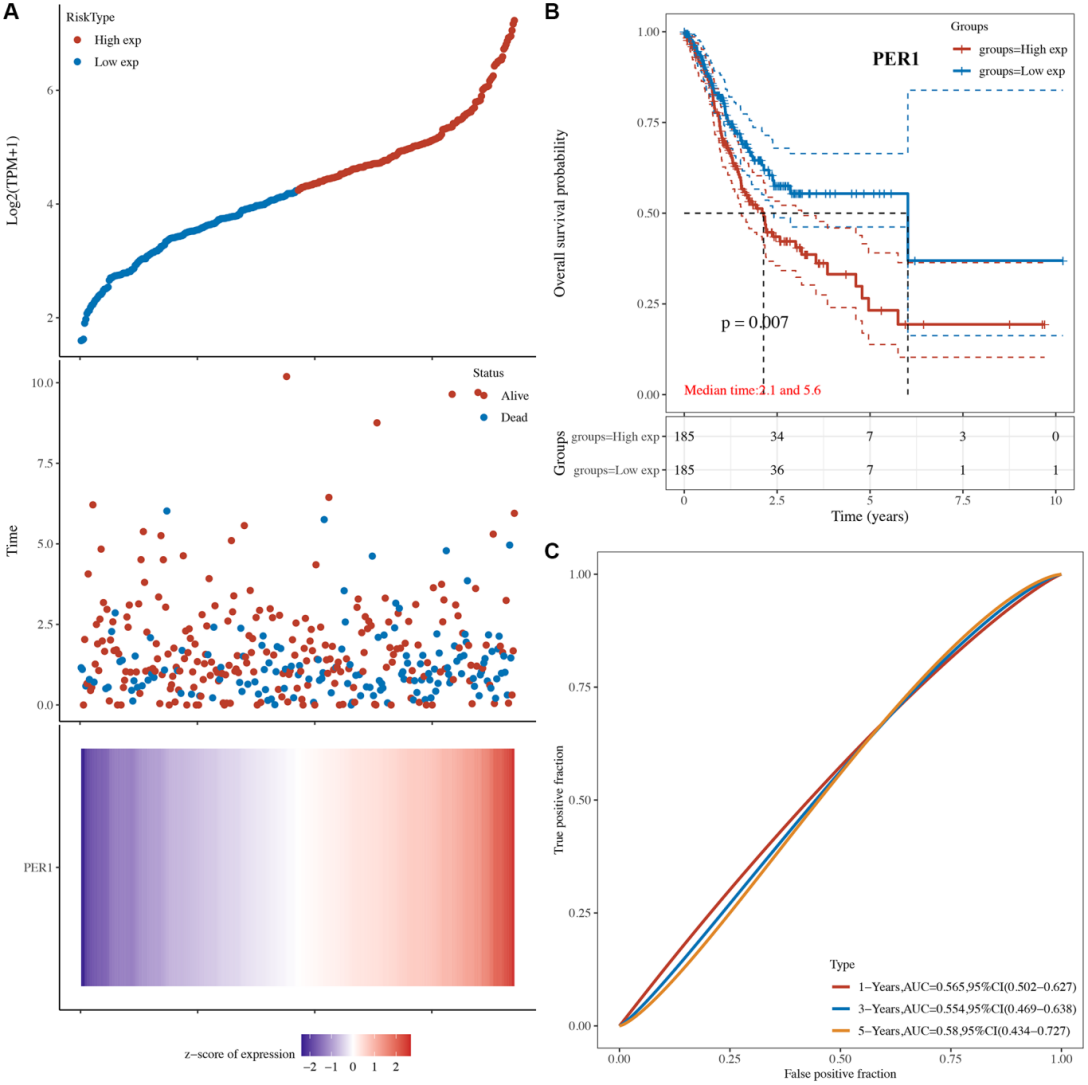
**The overall survival analysis of PER1 in STAD.** (**A**) The risk score, survival status and gene expression of each patient. (**B**) Kaplan-Meier overall survival curve of PER1 in STAD patients with high and low PER1 expression. (**C**) Time-dependent ROC of PER1 in predicting the prognosis of STAD patients. ROC receiver operating characteristic.

**Figure 6:**
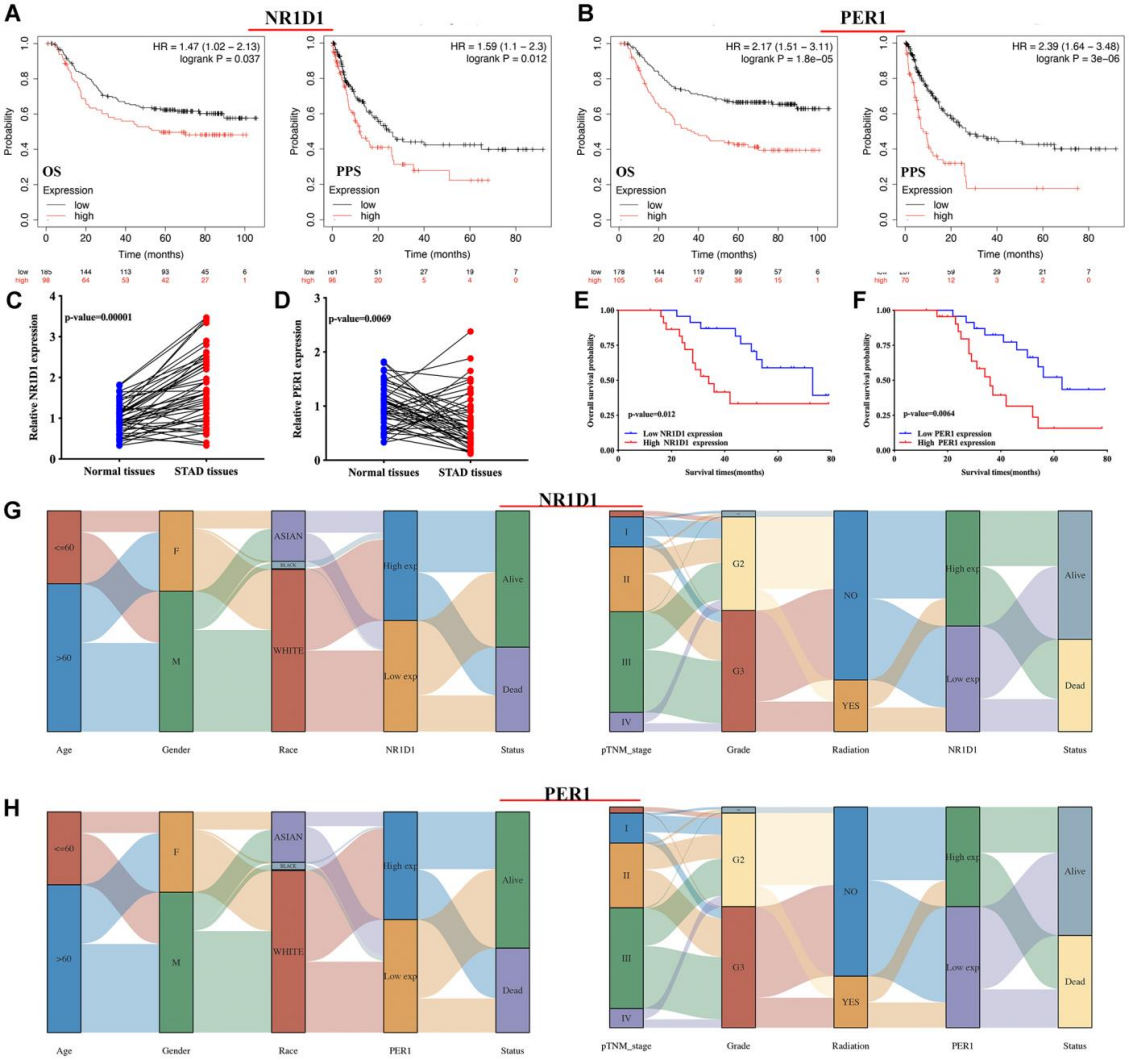
**The prognosis value and expression of core circadian clock genes in the subtypes of STAD tissues.** (**A**, **B**) The overall survival and progression-free survival curve of STAD patients with high/low NR1D1(A) and PER1(B) expression. (**C**, **D**) The relative expression of NR1D1 and PER1 in STAD tissues and normal tissues in clinical cohort. (**E**, **F**) The overall survival curve of STAD patients with high/low NR1D1 and PER1 expression in clinical cohort. (**G**) The expression of NR1D1 in the subtypes of STAD tissues. (**H**) The expression of PER1 in the subtypes of STAD tissues. Each column represents a characteristic variable, different colors represent different subtypes of STAD tissues, and lines represent the distribution of the same sample in different characteristic variables.

The risk score of core circadian genes in the TCGA STAD cohort was detected with Cox regression analysis. Kaplan-Meier curves were drawn on the basis of the risk score of each patient. The data of prognostic analysis of core circadian genes in STAD was shown in Table 1. Among these core circadian genes, the expression of PER1 and NR1D1 was linked to overall survival (OS), progression-free survival (PFS), and disease-free survival (DFS) rates in STAD patients. For the overall survival rate, the risk score of each patient is shown in Figure 4A and Figure 5A. STAD patients with high expression of NR1D1 (Figure 4B, *p* = 0.002, HR (95% CI) = 1.69 (1.21–2.36)) and PER1 (Figure 5B, *p* = 0.007, HR (95% CI) = 1.59 (1.14–2.22)) had poor OS rates with 5-year AUCs of 0.611 (Figure 4C) and 0.58 (Figure 5C), respectively. For the progression-free survival rate, the risk score of each patient is shown in Supplementary Figure 1A and Supplementary Figure 2A. STAD patients with high expression of NR1D1 (Supplementary Figure 1B, *p* = 0.008, HR (95% CI) = 1.62 (1.13–2.31)) and PER1 (Supplementary Figure 2B, *p* = 0.007, HR (95% CI) = 1.64 (1.14–2.35)) had poor PFS rates with 5-year AUCs of 0.615 (Supplementary Figure 1C) and 0.562 (Supplementary Figure 2C), respectively. For the disease-free survival rate analysis, the risk score of each patient is shown in Supplementary Figure 3A and Supplementary Figure 4A. STAD patients with high expression of NR1D1 (Supplementary Figure 3B, *p* = 0.002, HR (95% CI) = 1.92 (1.27–3.02)) and PER1 (Supplementary Figure 4B, *p* = 0.010, HR (95% CI) = 1.69 (1.10–2–60)) had poor DFS rates with 5-year AUCs of 0.642 (Supplementary Figure 3C) and 0.622 (Supplementary Figure 4C), respectively. To further verify the prognostic value of NR1D1 and PER2 in STAD, the GSE62254 dataset was obtained from the GEO database. The results indicated poor OS and PFS rates in STAD patients with high NR1D1 (Figure 6A, all *p* < 0.05) and PER2 (Figure 6B, all *p* < 0.05) expression. These results indicated that NR1D1 and PER2 serve as potential prognostic biomarkers in STAD.

**Table 1:** Prognosis value of core circadian genes in STAD.

**Genes**	**Overall Survival**		**Progression Free Survival**		**Disease Free Survival**	
	***p*-value**	**HR (95% CI)**	***p*-value**	**HR (95% CI)**	***p*-value**	**HR (95% CI)**
CLOCK	0.308	1.19 (0.85–1.65)	0.700	0.93 (0.66–1.33)	0.791	0.95 (0.62–1.43)
ARNTL	0.391	1.15 (0.83–1.60)	0.300	1.21 (0.85–1.72)	0.305	1.25 (0.82–1.89)
CRY1	0.207	0.81 (0.58–1.12)	0.634	0.92 (0.65–1.31)	0.689	0.92 (0.61–1.39)
CRY2	0.073	1.35 (0.97–1.89)	0.380	1.17 (0.82–1.67)	0.028	1.62 (1.05–2.49)
**NR1D1**	**0.002**	**1.69 (1.21–2.36)**	**0.008**	**1.62 (1.13–2.31)**	**0.002**	**1.96 (1.27–3.02)**
**PER1**	**0.007**	**1.59 (1.14–2.22)**	**0.007**	**1.64 (1.14–2.35)**	**0.010**	**1.69 (1.10–2.60)**
PER2	0.701	0.94 (0.68–1.30)	0.903	0.98 (0.69–1.39)	0.590	1.12 (0.74–1.71)
PER3	0.440	1.14 (0.82–1.58)	0.107	1.34 (0.94–1.92)	0.127	1.39 (0.91–2.12)
RORA	0.143	1.28 (0.92–1.77)	0.078	1.38 (0.96–1.96)	0.092	1.43 (0.94–2.18)

### Validation of the expression and prognostic value

Clinical tissues were used to further detect the mRNA level and prognostic significance of PER1/NR1D1 in STAD. Not surprisingly, NR1D1 expression was increased in STAD (Figure 6C, *p* = 0.00001) while PER1 expression was decreased in STAD tissues (Figure 6D, *p* = 0.0069) compared with that in normal tissues. Moreover, As shown in Figure 6E and Figure 6F, prognosis analysis suggested a poor overall survival in STAD patients with high NR1D1 expression (*p* = 0.012) and PER1 expression (*p* = 0.0064), These data further verified above results.

### The expression of NR1D1 and PER1 in STAD patient subtypes

The above results (result 3.4 and result 3.5) revealed that NR1D1 and PER1 may serve as prognostic biomarkers in STAD and are predictors of poor patient prognosis. Thus, NR1D1 and PER1 were selected for further analysis. We then analyzed the association between NR1D1/PER1 expression and clinicopathologic features. As shown in Figure 6G, STAD patients with high pTNM stage and high tumor grade had low NR1D1 expression compared with that of patients with low pTNM and low tumor grade. The PER1 results are presented in Figure 6H. Male STAD patients and patients with high tumor-grade had low PER1 expression.

### NR1D1/PER1 were correlated with immune infiltration in STAD

**Figure 7:**
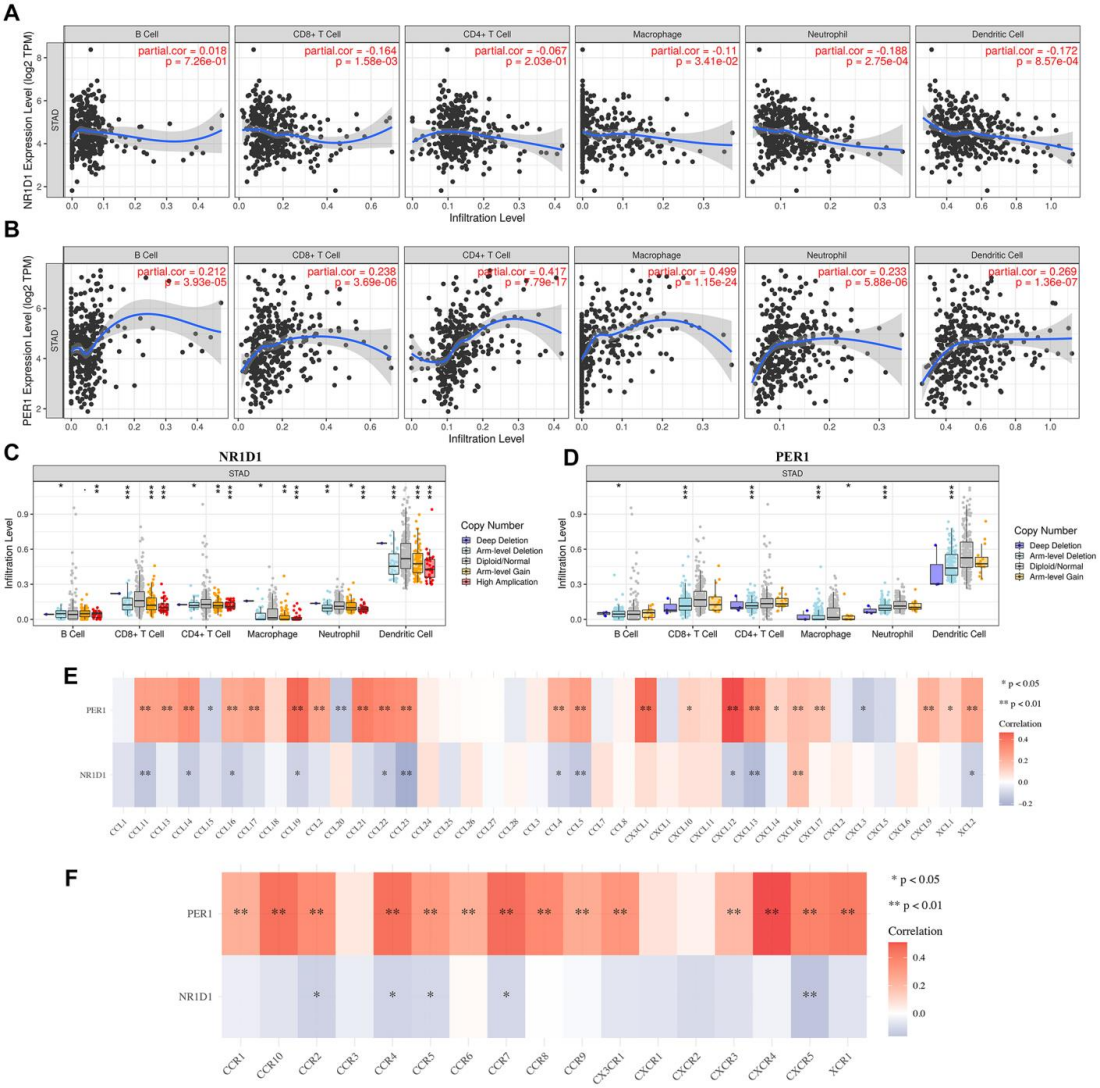
**The correlation between core circadian clock genes and immune infiltration (TIMER).** (**A**) The correlation between NR1D1 expression and the abundance of CD8+ T cells, CD4+ T cells, Macrophage, Neutrophils and Dendritic cells. (**B**) The correlation between PER1 expression and the abundance of CD8+ T cells, CD4+ T cells, Macrophage, Neutrophils and Dendritic cells. (**C**) the correlation between SCNA of NR1D1 and immune cell infiltration. (**D**) the correlation between SCNA of PER1 and immune cell infiltration. (**E**) the correlation between core circadian clock genes and the expression of chemokines in STAD. (**F**) the correlation between core circadian clock genes and the expression of chemokine receptors in STAD. Red color represents positive correlation, blue color represents negative correlation. SCNA, somatic copy number alterations; ^*^P < 0.05, ^**^P < 0.01, ^***^P < 0.001.

Immune infiltration is a vital factor affecting sentinel lymph node status and prognosis in cancers [12–14]. Recent evidences have elaborated the close correlation between circadian rhythm and lung cancer [5]. To identify whether core circadian genes could act as immunotherapy targets for STAD, we first clarified the correlation between the mRNA level of NR1D1/PER1 and immune infiltration in STAD. We found that the mRNA level of NR1D1 was negatively associated with the abundance of CD8+ T cells (Cor = –0.164, *p* = 1.58E-3), macrophages (Cor = –0.11, *p* = 3.41E-2), neutrophils (Cor = –0.188, *p* = 2.75E-4), and dendritic cells (Cor = –0.172, *p* = 8.57E-4) (Figure 7A). PER1 expression was positively correlated with the abundance of B cells (Cor = 0.212, *p* = 3.39E-5), CD8+ T cells (Cor = 0.238, *p* = 3.69E-6), CD4+ T cells (Cor = 0.417, *p* = 7.79E-17), macrophages (Cor = 0.499, *p* = 1.15E-24), neutrophils (Cor = 0.233, *p* = 5.88E-6) and dendritic cells (Cor = 0269, *p* = 1.36E-7) (Figure 7B). Moreover, we found that somatic copy number alteration (SCNA) of NR1D1 (Figure 7C) and PER1 (Figure 7D) could partially suppress immune infiltration in STAD.

Additionally, we sought to calculate the association between NR1D1/PER1 expression and immune cell markers. As shown in Table 2, NR1D1 expression was correlated with certain biomarkers of these immune cells, while PER1 expression showed a positive correlation with most of the biomarkers of these immune cells (Table 2). Chemokines and their receptors modulate immune surveillance in oncogenesis, metastasis, and response to immunotherapy [15]. In our study, the correlation analysis demonstrated a strong correlation between PER1/NR1D1 and chemokines as well as chemokine receptors (Figure 7E–7F). These data demonstrated the possible correlation between the circadian clock and immune infiltrates in STAD.

**Table 2:** Correlation analysis between NR1D1/PER1 and gene biomarkers of immune cells in STAD.

**Description**	**Gene markers**	**STAD**			
		**NR1D1**		**PER1**	
		**Cor**	***P*-value**	**Cor**	***P*-value**
CD8+ T cell	CD8A CD8B	–0.139 –0.063	^**^ 0.203	0.331 0.255	^***^ ^***^
T cell (general)	CD3D CD3E CD2	–0.213 –0.13 –0.177	^***^ ^**^ ^***^	0.256 0.31 0.277	^***^ ^***^ ^***^
B cell	CD19 CD79A	–0.113 –0.158	^*^ ^**^	0.366 0.366	^***^ ^***^
Monocyte	CD86 CD115(CSF1R)	–0.133 –0.077	^**^ 0.119	0.305 0.414	^***^ ^***^
TAM	CCL2 CD68 IL10	–0.076 0.079 –0.102	0.121 0.107 ^*^	0.249 0.198 0.29	^***^ ^***^ ^***^
M1 Macrophage	INOS (NOS2) IRF5 COX2(PTGS2)	–0.016 0.101 0.016	0.74 ^*^ 0.745	–0.057 0.224 0.173	0.245 ^***^ ^***^
M2 Macrophage	CD163 VSIG4 MS4A4A	–0.043 –0.081 –0.13	0.379 0.0994 ^**^	0.405 0.335 0.393	^***^ ^***^ ^***^
Neutrophils	CD66b (CEACAM8) CD11b (ITGAM) CCR7	0.048 –0.022 –0.104	0.325 0.659 ^*^	0.006 0.407 0.449	0.905 ^***^ ^***^
Natural killer cell	KIR2DL1 KIR2DL3 KIR2DL4 KIR3DL1 KIR3DL2 KIR3DL3 KIR2DS4	–0.176 –0.129 –0.158 –0.189 –0.168 0.035 –0.167	^***^ ^**^ ^**^ ^***^ ^***^ 0.478 ^***^	0.142 0.072 0.079 0.171 0.198 0.058 0.112	^**^ 0.143 0.106 ^***^ ^***^ 0.24 ^*^
Dendritic cell	HLA-DPB1 HLA-DQB1 HLA-DRA HLA-DPA1 BDCA-1(CD1C) BDCA-4(NRP1) CD11c (ITGAX)	–0.119 –0.065 –0.112 –0.066 –0.163 0.01 –0.053	^*^ 0.185 ^*^ 0.179 ^***^ 0.839 0.282	0.321 0.238 0.243 0.29 0.382 0.457 0.344	^***^ ^***^ ^***^ ^***^ ^***^ ^***^ ^***^
Th1	T-bet (TBX21) STAT4 STAT1 IFN-g (IFNG) TNF-a (TNF)	–0.12 –0.167 0.063 –0.081 –0.002	^*^ ^***^ 0.203 0.0975 0.974	0 .307 0.418 0.15 0 0.126	^***^ ^***^ ^**^ 0.996 ^*^
Th2	GATA3 STAT6 STAT5A IL13	–0.082 0.18 –0.008 0.017	0.0934 ^***^ 0.877 0.723	0.344 0.227 0.376 0.059	^***^ ^***^ ^***^ 0.232
Tfh	BCL6 IL21	0.081 –0.163	0.0996 ^***^	0.571 0.096	^***^ ^*^
Th17	STAT3 IL17A	0.102 0.068	^*^ 0.167	0.445 –0.164	^***^ ^***^
Treg	FOXP3 CCR8 STAT5B TGFb (TGFB1)	–0.037 –0.034 0.065 0.039	0.448 0.487 0.185 0.423	0.305 0.354 0.45 0.455	^***^ ^***^ ^***^ ^***^
T cell exhaustion	PD-1 (PDCD1) CTLA4 LAG3 TIM-3 (HAVCR2) GZMB	–0.085 –0.066 –0.114 –0.102 –0.111	0.0831 0.179 ^*^ ^*^ ^*^	0.278 0.238 0.229 0.309 0.098	^***^ ^***^ ^***^ ^***^ ^*^

^*^p < 0.05, ^**^p < 0.01, ^***^p < 0.001.

## DISCUSSION

As a vital regulator of human physiology, the circadian clock exerted a vital function in the regulation of oscillations in biological processes and behaviors [16]. Increasing data have suggested that circadian disruption is an independent risk factor for cancer [17, 18]. Moreover, previous study indicated certain core circadian clock genes as prognostic biomarkers in cancers [19]. However, the specific functions of circadian clock in the prognosis and therapy of STAD is still unclear. Therefore, the aim of this study was to clarify the role of the circadian clock in STAD using multiomics tools.

The expression of core circadian clock genes could largely define the circadian clock state [20]. We first detected the expression of core circadian clock genes in STAD. The results suggested upregulation of NR1D1, CLOCK and CRY1, while the mRNA levels of CRY2, PER1, PER3 and RORA were downregulated in STAD versus gastric tissues. The upregulated genes (CLOCK, CRY1 and NR1D1) in the STAD microenvironment were defined as the “daytime” stage, and the downregulated genes (CRY2, PER1, PER3 and RORA) in the STAD microenvironment were defined as the “nighttime” stage. Under the regulation of these altered core circadian clock genes, circadian rhythms were disrupted, which might contribute to the carcinogenesis and progression of STAD. Therefore, investigating the STAD-specific circadian clock may be a potential strategy for identifying anticancer drugs.

Interestingly, our results showed that these genes were linked to the activity of certain oncogenic pathways, including the apoptosis and cell cycle pathway. A previous study revealed cross-talk between the circadian clock and the cell cycle as well as apoptosis in cancer [21]. The results of another study suggested that circadian clock and cell cycle systems are robustly phase-coupled in a bidirectional manner [22]. In our study, core circadian clock genes had an inhibitory effect on the apoptosis pathway, cell cycle pathway, and DNA damage response pathway, which may promote the carcinogenesis and progression of STAD. This finding further confirmed circadian disruption as an independent risk factor for cancer [17].

Another crucial result of the current study was that NR1D1 and PER1 served as potential prognostic markers in STAD and are associated with poor prognosis in STAD patients, including OS, PFS and DFS rates. Univariate and multivariate analyses demonstrated that NR1D1, PER1, pT stage, and pM stage were independent factors affecting the prognosis of STAD patients. In addition to STAD, core circadian clock genes were suggested to be prognostic markers in other malignancies. In breast cancer patients with chemotherapy, NR1D1 is a prognostic marker predicting good prognosis [23]. Moreover, lower mRNA of PER1, PER2 and PER3 are linked to poor overall survival rates in lung cancer patients, indicating that these genes are prognostic markers [24]. Bo et al. suggested that CLOCK is a biomarker in hepatocellular carcinoma that predicts poor prognosis [25].

Interestingly, our study also revealed a conspicuous pertinence between the level of NR1D1/PER1 and immune cells, immune biomarkers, chemokines and chemokine receptors. NR1D1 and PER1 were significantly linked to the immune infiltration level of immune cells. A previous study found that CD4(+) T helper (T(H)17) cell differentiation is regulated by the circadian clock [26]. Our result was also inconsistent with the data of a previous study showing that circadian expression of clock genes was linked to the infiltration level of macrophages, dendritic cells, and B cells [26]. Our study also revealed associations between core circadian clock genes and immune biomarkers, chemokines and chemokine receptors, which play a vital role in the immune response and tumor microenvironment. These data demonstrated the possible association between the circadian clock and immune infiltrates in STAD.

There is no doubt that our study had some limitations. First, STAD-related core circadian clock gene information was only retrieved from the literature, and the information obtained may not be comprehensive. Moreover, it would be beneficial to verify our results with *in vitro* and *in vivo* experiments.

Overall, in this study, a comprehensive analysis of the prognostic value and immune-related function of core circadian clock genes in STAD was performed, and our results suggested that NR1D1 and PER1 serve as prognostic biomarkers and are associated with immune infiltration in STAD.

### Consent for publication

Our research was approved and supported by an ethics committee of the Second Affiliated Hospital of Nanchang University. All patients provided informed consent.

### Availability of data and materials

The analyzed data sets generated during the study are available from the corresponding author on reasonable request.

## Supplementary Materials

Supplementary Figures

Supplementary Table 1
