# Supplementary material for: The circadian clock is associated with prognosis and immune infiltration in stomach adenocarcinoma
Source: Aging (Albany NY). 2021 Jun 23;13(12):16637–55. doi: 10.18632/aging.203184 (PMC8266362; doi:10.18632/aging.203184)
Supplement: Supplementary Figures [file aging-13-203184-s001.pdf]

SUPPLEMENTARY FIGURES

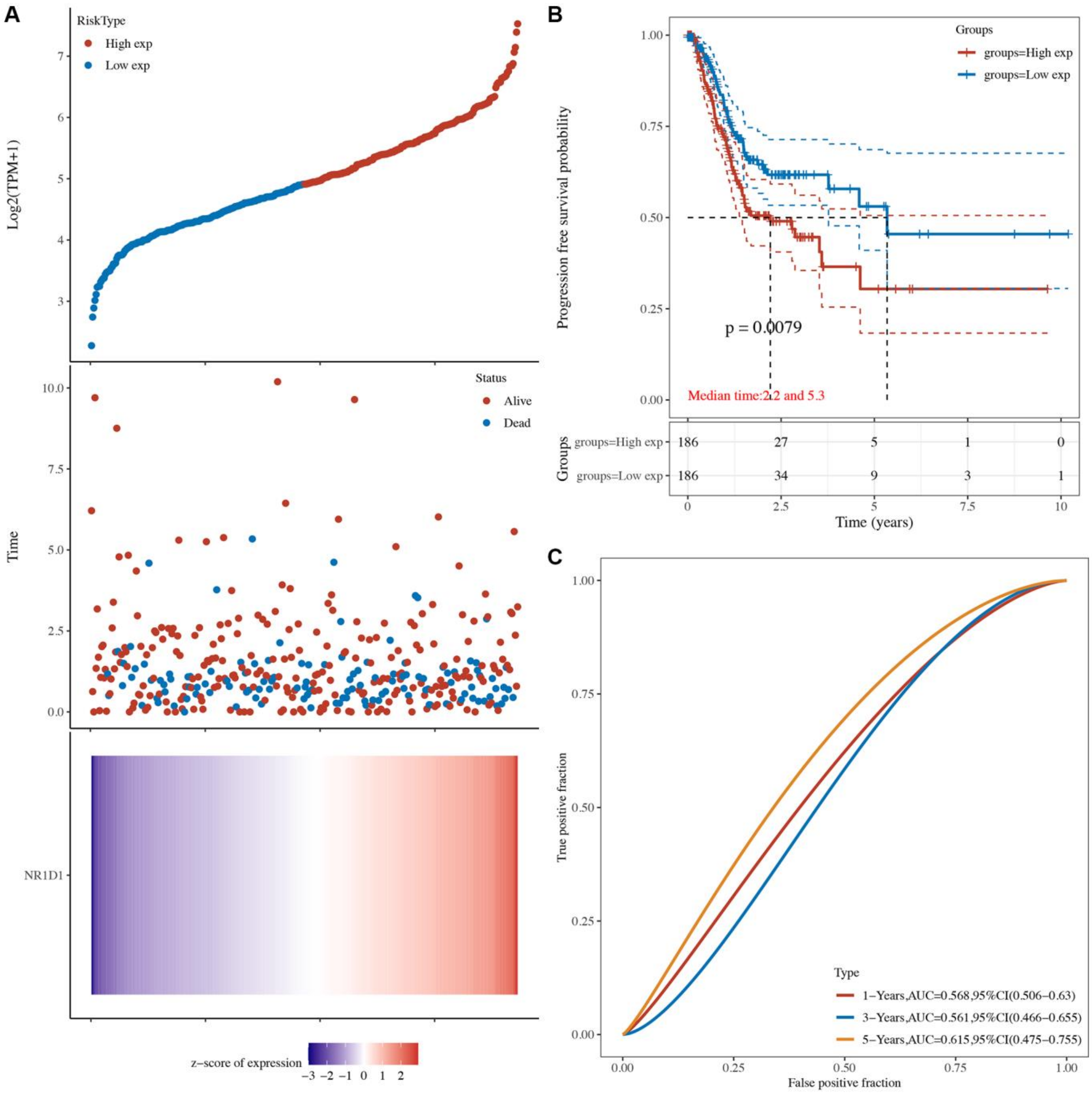

Supplementary Figure 1. The progression free survival analysis of NR1D1 in STAD. (**A**) The risk score, survival status and gene expression of each patient. (**B**) Kaplan-Meier progression free survival curve of NR1D1 in STAD patients with high and low NR1D1 expression. (**C**) Time-dependent ROC of P NR1D1 in predicting the prognosis of STAD patients. ROC receiver operating characteristic.

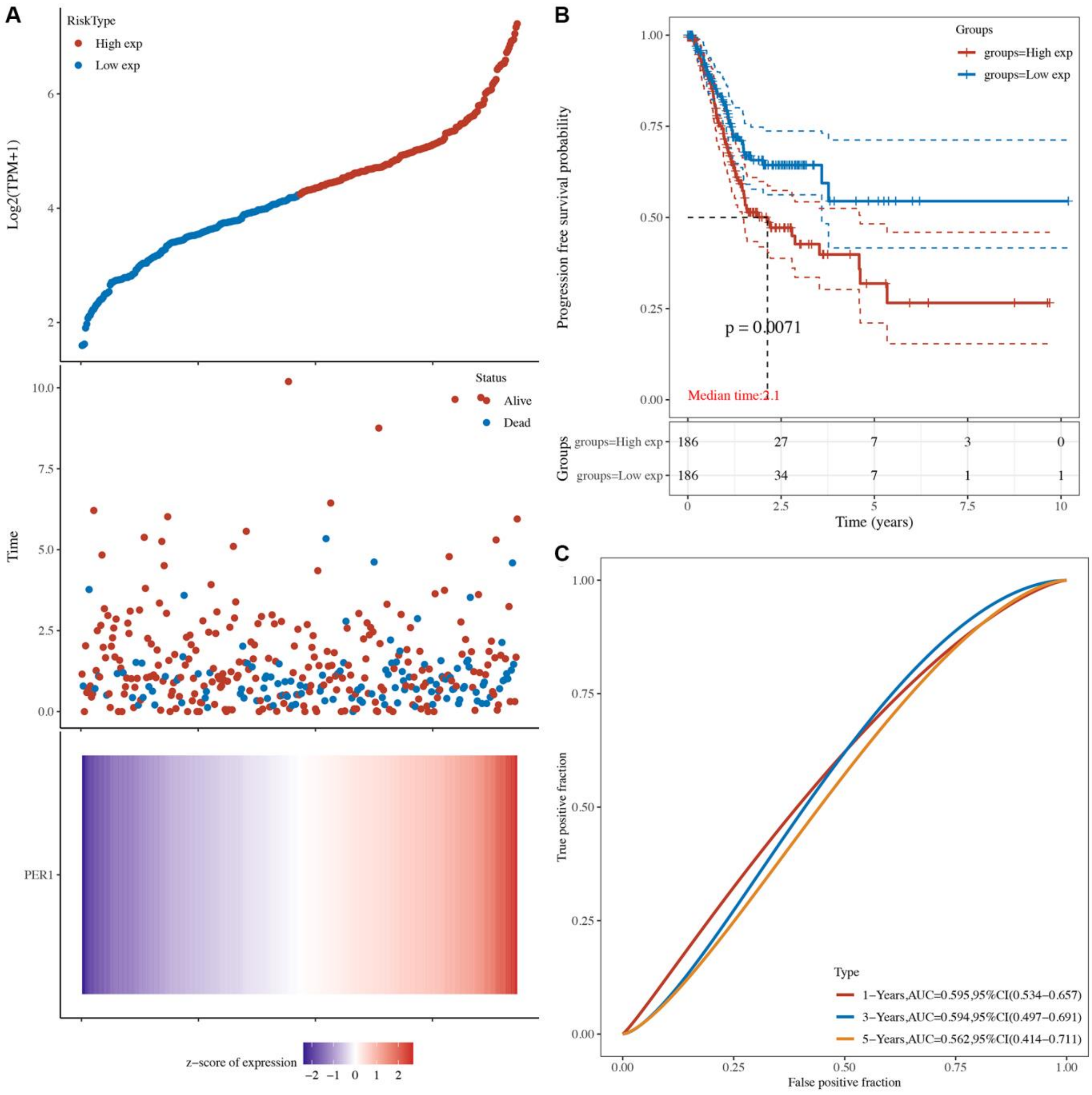

Supplementary Figure 2. The progression free survival analysis of PER1 in STAD. (**A**) The risk score, survival status and gene expression of each patient. (**B**) Kaplan-Meier progression free survival curve of PER1 in STAD patients with high and low PER1 expression. (**C**) Time-dependent ROC of PER1 in predicting the prognosis of STAD patients. ROC receiver operating characteristic.

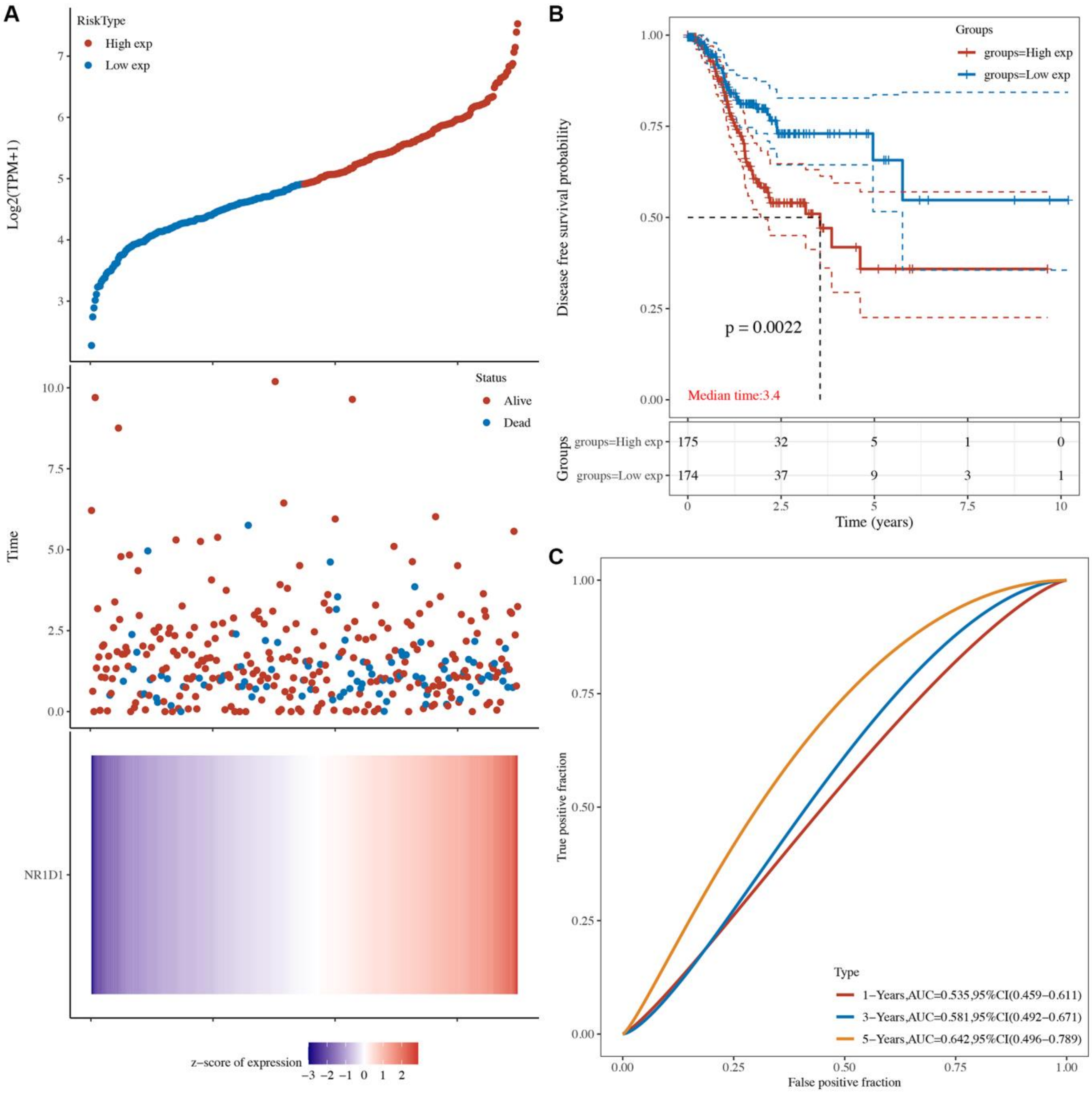

Supplementary Figure 3. The disease-free survival analysis of NR1D1 in STAD. (**A**) The risk score, survival status and gene expression of each patient. (**B**) Kaplan-Meier disease free survival curve of NR1D1 in STAD patients with high and low NR1D1 expression. (**C**) Time-dependent ROC of NR1D1 in predicting the prognosis of STAD patients. ROC receiver operating characteristic.

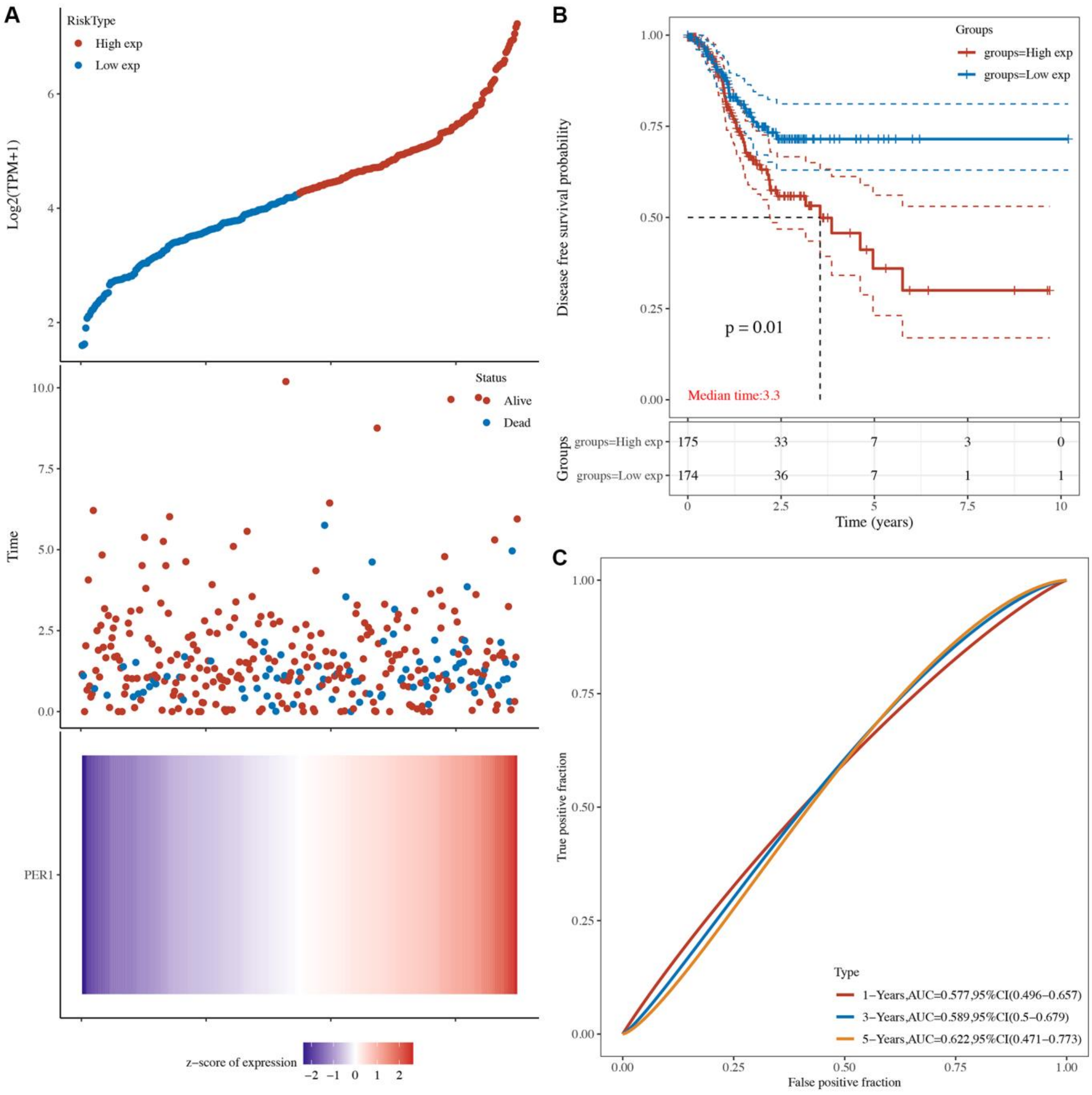

Supplementary Figure 4. The disease-free survival analysis of PER1 in STAD. (**A**) The risk score, survival status and gene expression of each patient. (**B**) Kaplan-Meier disease free survival curve of PER1 in STAD patients with high and low PER1 expression. (**C**) Time-dependent ROC of PER1 in predicting the prognosis of STAD patients. ROC receiver operating characteristic.
