# Supplementary material for: The circadian clock is associated with prognosis and immune infiltration in stomach adenocarcinoma
Source: Aging (Albany NY). 2021 Jun 23;13(12):16637–55. doi: 10.18632/aging.203184 (PMC8266362; doi:10.18632/aging.203184)
Supplement: Supplementary Table 1 [file aging-13-203184-s002.pdf]

SUPPLEMENTARY TABLE

Supplementary Table 1. Primer sequences for reverse transcription-quantitative polymerase chain reaction.

Genes	Forward (5′–3′)	Reverse (5′–3′)
GAPDH	GCACCGTCAAGGCTGAGAAC	TGGTGAAGACGCCAGTGGA
PER1	GGACATGACCTCTGTGCTGA	CATCAGGGTGACCAGGATCT
NR1D1	GCTAGACAGCCAGACAGCTAG	AGCGTCGCCACTGTACATGAGC
